# Small Bowel Gastrointestinal Stromal Tumors: A 15-Year Cohort Study Focusing on Jejuno-Ileal Site-Specific Outcomes and Prognostic Factors

**DOI:** 10.3390/cancers18020218

**Published:** 2026-01-09

**Authors:** Yuichi Kojima, Kentaro Tominaga, Yuzo Kawata, Chizuru Kaneko, Shuhei Kondo, Yoshifumi Shimada, Junji Yokoyama, Toshifumi Wakai, Shuji Terai

**Affiliations:** 1 Division of Gastroenterology and Hepatology, Graduate School of Medical and Dental Sciences, Niigata University, Niigata 9518510, Japan; y-kojima@med.niigata-u.ac.jp (Y.K.);; 2 Division of Molecular and Diagnostic Pathology, Niigata University Graduate School of Medical and Dental Sciences, Niigata University, Niigata 9518510, Japan; 3 Division of Digestive and General Surgery, Graduate School of Medical and Dental Sciences, Niigata University, Niigata 9518510, Japan; 4 Department of Gastroenterology, Saiseikai Niigata Hospital, Niigata 9501104, Japan

**Keywords:** gastrointestinal stromal tumors, Ki-67, neurofibromatosis type 1, small bowel, jejuno-ileal, prognostic factors

## Abstract

**Background:** Site-specific long-term outcomes, including neurofibromatosis type 1 (NF1), Ki-67 prognostic value, and very late recurrences of small bowel gastrointestinal stromal tumors (GISTs), remain inadequately defined. **Methods:** This retrospective cohort study investigated the clinical characteristics, diagnostic challenges, and long-term outcomes of patients with small bowel GISTs. This retrospective, single-center study (2008–2024) analyzed 27 consecutive patients (average age: 62.2 years) with jejunal/ileal GISTs. Clinicopathologic features, diagnostic yield of balloon-assisted enteroscopy (BAE), treatments, and outcomes were evaluated during a 10.2-year median follow-up period. Recurrence-free survival (RFS) and overall survival (OS) were estimated by Kaplan–Meier with log-rank testing. Ki-67 was assessed using MIB-1; a prespecified 5% cut-off was chosen based on prior evidence. **Results:** Tumor (mean size, 62.4 mm) sites included the jejunum (74.1%) and ileum (25.9%). NF1 was present in 3/27 (11.1%) patients, all with multiple jejunal tumors. Among the 14 patients who underwent BAE, biopsy was attempted in six and yielded a histological diagnosis in one (16.7%). Six patients had recurrence; two died from disease >10 years postoperatively. Five-year OS and RFS were 91.3% and 68.7%, respectively. Adverse RFS was associated with ileal location (*p* = 0.03), size ≥ 10 cm (*p* < 0.001), mitoses > 5/50 high-power fields (*p* = 0.002), and Ki-67 ≥ 5% (*p* < 0.001). One patient labeled low risk by conventional models had recurrence with Ki-67 = 10%. Another classified as low risk by conventional models experienced recurrence >10 years after surgery, with a Ki-67 index of 10%. **Conclusions:** Extended, risk-adapted surveillance may be reasonable for small-bowel GISTs, and it may be beneficial to incorporate Ki-67 (≥5%) into site-based risk stratification. These observations remain hypothesis-generating and require validation in larger, multicenter cohorts and prospective studies.

## Simple Summary

Small bowel gastrointestinal stromal tumors (GISTs) are rare; however, they are more aggressive than those in the stomach. Preoperative diagnosis is often challenging due to their submucosal origin, leading to diagnostic delay. This study analyzed 27 patients with tumors in the jejunum or ileum for 15 years to clarify long-term outcomes and risk factors. We found that tumors in the ileum, larger than 10 cm, and with high cell division or Ki-67 index were more likely to recur. Some patients developed recurrence more than 10 years after surgery, even when initially considered to have low risk. We also observed that patients with neurofibromatosis type 1 often had multiple tumors. Our findings indicate that prolonged follow-up may be warranted for selected patients and that Ki-67 could serve as an additional marker in risk assessment. These results are hypothesis-generating and should be interpreted with caution given the small sample size.

## 1. Introduction

Gastrointestinal stromal tumors (GISTs), which originate from the interstitial cells of Cajal or their precursors, are the most common mesenchymal neoplasms of the gastrointestinal tract. Although gastric GISTs account for approximately 60–70% cases, the small intestine is the second most frequent site, comprising 20–30% of all GISTs. Importantly, small bowel GISTs have a higher malignant risk and poorer prognosis than do their gastric counterparts, even after complete surgical resection [1,2].

Recent advances in diagnostic modalities, including capsule endoscopy and balloon-assisted enteroscopy, have improved visualization of small-bowel lesions. However, preoperative histological confirmation remains difficult because of the submucosal nature of these tumors and bleeding risk, often resulting in delayed diagnosis and presentation of large tumors or complications such as gastrointestinal bleeding [3,4].

Neurofibromatosis type 1 (NF1) is a recognized risk factor for GIST development, particularly in the small intestine where tumors frequently present as multiple lesions. Although NF1-associated GISTs have been reported, their clinical behavior and long-term outcomes remain poorly defined because of their rarity [5,6].

In addition, long-term outcomes of small bowel GISTs, including late recurrence beyond 10 years and behavior of tumors classified as low risk, are not well documented. This lack of information hampers establishment of optimal surveillance strategies. The diagnostic yield of balloon-assisted enteroscopy in real-world settings and role of adjuvant therapy in this subset also require further clarification.

In this study, we retrospectively analyzed 27 patients with small bowel GISTs (excluding duodenal tumors) diagnosed and treated at a single institution during a 15-year period. Our aim was to elucidate the clinical characteristics, diagnostic challenges, and long-term outcomes of these tumors, with particular focus on NF1-associated cases, the limitations of enteroscopic biopsy, and late recurrence incidences. We also evaluated the prognostic significance of the Ki-67 labeling index, which may complement traditional risk stratification systems [7]. Although large dataset analyses have suggested minimal differences in survival between duodenal and jejuno-ileal GISTs [8,9], we excluded duodenal tumors to reduce heterogeneity and focus on jejuno-ileal tumors, which often present distinct diagnostic and therapeutic considerations and reportedly show greater malignant potential than do gastric GISTs. This study was designed not only as a descriptive analysis but also as an investigation into the biological and clinical drivers of the known aggressiveness of small bowel GISTs. We hypothesized that site-specific factors, such as microenvironmental differences and proliferative activity reflected by Ki-67, may contribute to the higher malignant potential observed in jejuno-ileal tumors compared with gastric counterparts.

## 2. Materials and Methods

### 2.1. Study Design and Patients

**Figure 1:**
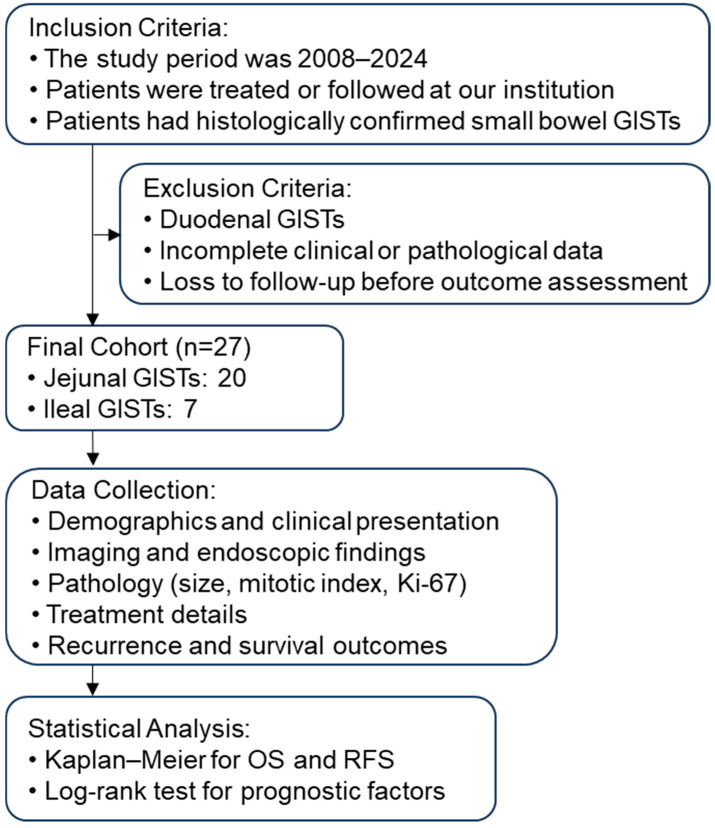
Flowchart illustrating patient selection, inclusion/exclusion criteria, and the study process. Patients with histologically confirmed small bowel GISTs treated or followed at our institution between 2008 and 2024 were screened. The inclusion criteria were jejunal or ileal location and complete clinical data. Duodenal GISTs and cases with incomplete data were excluded. The final cohort comprised 27 patients. Data collection included demographics, clinical presentation, imaging and endoscopic findings, pathological features (size, mitotic index, Ki-67), and treatment details. Follow-up was performed to assess recurrence and survival outcomes. Statistical analysis was performed using Kaplan–Meier methods for overall survival (OS) and recurrence-free survival (RFS), with log-rank tests for prognostic factors. GIST, gastrointestinal stromal tumor; OS, overall survival; RFS, recurrence-free survival.

Patients with histologically confirmed small bowel GISTs (excluding duodenal tumors) treated or followed up at our institution between 2008 and 2024 were included. Patients with duodenal GIST were excluded because these tumors are anatomically close to the stomach, often detected by standard upper endoscopy, and generally exhibit different prognostic characteristics compared with jejuno-ileal GISTs. The patient selection process is summarized in Figure 1.

The Ki-67 labeling index was assessed using the MIB-1 monoclonal antibody. Hot spot areas were selected, and at least 500 tumor cells were counted under high-power fields (HPFs). The percentage of positive nuclei was calculated as follows: a cutoff of 5% was adopted based on prior meta-analyses and studies demonstrating its prognostic relevance in GIST [7,8]. The Ki-67 labeling index was assessable in 22 patients (81.5%). Five patients lacked evaluable tissue due to archival block depletion.

### 2.2. Inclusion and Exclusion Criteria

Patients were eligible if they met all the inclusion criteria: (1) histologically confirmed small bowel GIST, (2) jejunal or ileal location, and (3) treatment or follow-up at our institution between 2008 and 2024.

Patients with a duodenal GIST or incomplete clinical or pathological data and those who were lost to follow-up before outcome assessment were excluded.

### 2.3. Data Collection

Clinical data were obtained from electronic medical records, including demographic characteristics (age, sex); presenting symptoms; comorbidities (including NF1); and tumor location, size, and number of lesions. Diagnostic modalities, including imaging results and endoscopic procedures, were reviewed. For patients who underwent balloon-assisted enteroscopy, the success of lesion visualization and histological confirmation was recorded. All images were obtained from the institutional database following acquisition of written patient consent.

### 2.4. Pathological Assessment

Histopathological diagnosis was based on hematoxylin and eosin staining and immunohistochemistry for KIT (CD117), DOG1, and CD34. Risk stratification was performed using the Miettinen and modified Fletcher classifications [9,10,11], considering tumor size, mitotic index, and anatomical site.

### 2.5. Treatment and Follow-Up

Surgical procedures, use of adjuvant imatinib, and subsequent treatments were documented.

The follow-up data included recurrence, metastasis, and survival outcomes. Recurrence was defined as radiologically or histologically confirmed local or distant disease after initial curative resection. Follow-up duration was calculated from the surgery date to the last clinical visit during this study period or death.

### 2.6. Statistical Analyses

Descriptive statistics were used to summarize patient characteristics and outcomes. Continuous variables are expressed as means with ranges and categorical variables as counts and percentages. A Kaplan–Meier survival analysis was performed to estimate overall survival (OS) and recurrence-free survival (RFS), and differences between groups were assessed using the log-rank test. Given the limited number of events, we prespecified a parsimonious Cox proportional hazards model including tumor size ≥ 10 cm and Ki-67 ≥ 5% and conducted complete case analysis for Ki-67 to identify independent prognostic factors for RFS. A sensitivity analysis added ileal location. Hazard ratios (HRs) with 95% confidence intervals (CIs) were estimated using Breslow ties. The sensitivity analysis assuming missing values as <5% or ≥5% did not alter the direction of the association. We also acknowledge that adjuvant imatinib therapy administered to high-risk patients may have influenced recurrence-free survival and, consequently, the interpretation of “natural” recurrence risk. All statistical analyses were performed using IBM SPSS Statistics version 28.0 (IBM Corp., Armonk, NY, USA).

## 3. Results

### 3.1. Patient Characteristics

A total of 27 patients with small bowel GISTs (excluding the duodenum) were included. Their mean age was 62 (range 25–86) years, with a slight female predominance (16 women, 11 men). Tumors were located in the jejunum in 20 patients (74.1%) and ileum in seven patients (25.9%). Twenty patients (74.1%) were symptomatic at diagnosis, most commonly presenting with gastrointestinal bleeding (*n* = 12), followed by abdominal pain including ileus (*n* = 4), abdominal distension (*n* = 3), and a palpable mass (*n* = 1). Seven patients (25.9%) were asymptomatic. A history of other malignancies was present in seven patients (25.9%). NF1 was identified in three patients (11.1%), all of whom had multiple jejunal tumors (Table 1).

### 3.2. Diagnostic Modalities

Contrast-enhanced computed tomography (CT) was performed for 24 patients (88.8%), magnetic resonance imaging (MRI) for two (7.4%), and capsule endoscopy for one (3.7%). Fourteen (51.9%) patients underwent balloon-assisted enteroscopy and biopsy was attempted in six of them; however, histological GIST confirmation was achieved in only one patient. The mean tumor size was 62.4 mm (range 13–145 mm). At diagnosis, distant metastases were present in two patients (7.4%), involving the liver (*n* = 1) and peritoneum (*n* = 1) (Table 1).

### 3.3. Representative Patient with a Preoperative Histological Diagnosis

**Figure 2:**
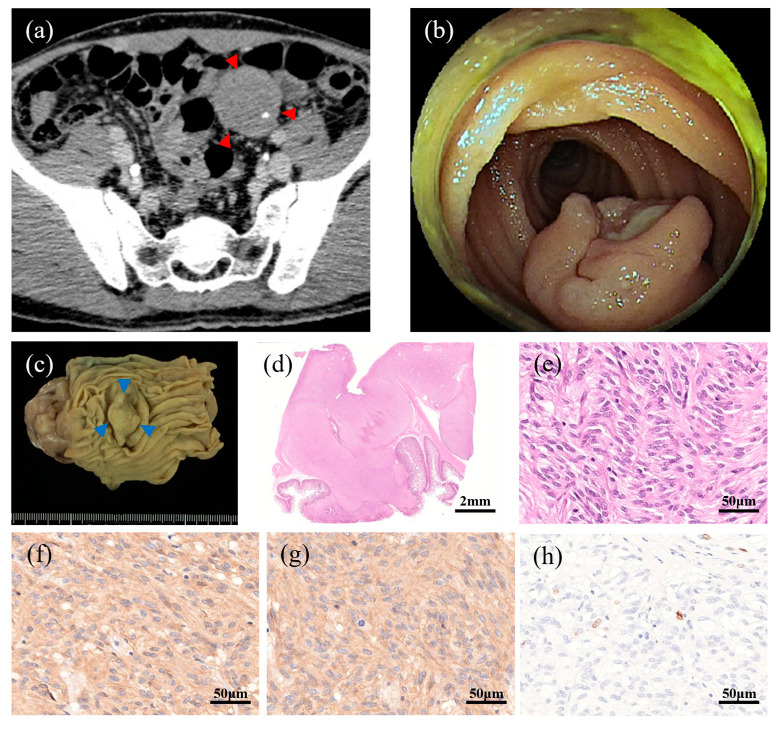
Representative case with a preoperative histological diagnosis of small bowel GIST. (**a**) Contrast-enhanced CT showing a well-demarcated mass in the ileum with faint enhancement, highlighted by red arrowheads. (**b**) Double-balloon enteroscopy image showing a submucosal tumor with central ulceration. (**c**) Gross appearance of the resected specimen showing a well-demarcated solid mass, highlighted by blue arrow heads. (**d**) Low-power view of HE (×10). (**e**) High-power HE (×400) image demonstrating elongated spindle cells with eosinophilic cytoplasm. (**f**) Immunohistochemical staining for KIT (CD117) showing diffuse cytoplasmic positivity. (**g**) DOG1 immunostaining showing membranous positivity. (**h**) Ki-67 immunostaining highlighting proliferative activity in hot spots.

Among the six patients who underwent biopsy during balloon-assisted enteroscopy, only 1 obtained a definitive histological GIST diagnosis (Table 1). The corresponding imaging and pathological findings for this representative patient are presented in Figure 2. Figure 2a shows the contrast-enhanced CT image revealing a well-demarcated, slightly enhanced mass in the ileum. Figure 2b shows the endoscopic view obtained via double-balloon enteroscopy, which allowed direct visualization and targeted biopsy of the submucosal tumor. The histopathological findings before surgical resection confirmed the diagnosis of GIST with positive immunostaining for KIT and DOG1. The resected specimen demonstrated a well-circumscribed solid tumor measuring 15 mm in diameter (Figure 2c). The histological examination revealed spindle cells arranged in interlacing fascicles, consistent with typical GIST morphology (Figure 2d,e). Immunohistochemistry showed diffuse positivity for KIT (CD117) and DOG1 (Figure 2f,g), confirming the GIST diagnosis. Ki-67 immunostaining revealed focal areas of increased proliferative activity (“hot spots”), which supported the assessment of tumor aggressiveness (Figure 2h).

### 3.4. Surgical and Adjuvant Treatments

**Figure 3:**
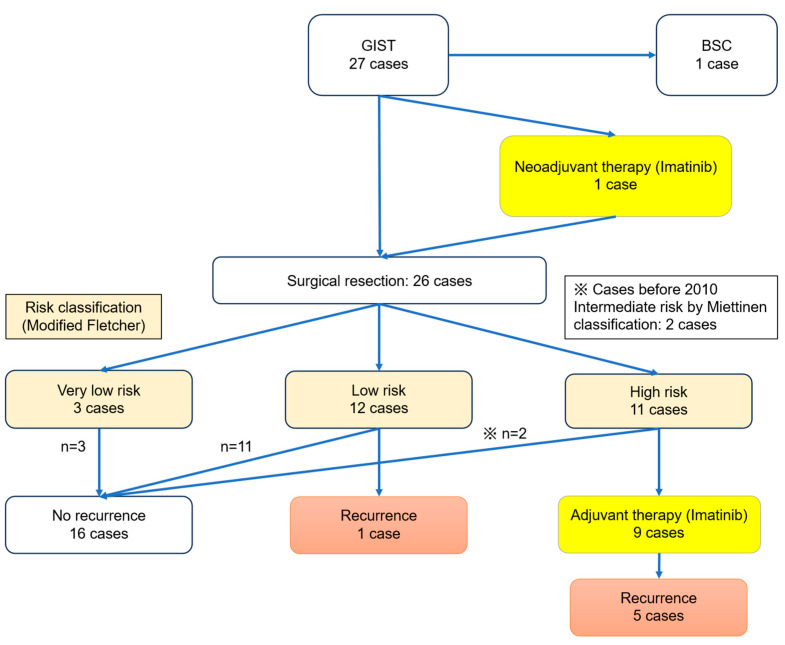
Risk classification and adjuvant therapy for 27 patients with small bowel GISTs. Distribution of risk categories according to Miettinen and modified Fletcher classifications and the number of patients who received adjuvant imatinib therapy. BSC, best supportive care; GIST, gastrointestinal stromal tumor.

Of the 27 patients, 26 underwent surgical resection of the primary tumor; one patient with multiple liver metastases and poor performance status received best supportive care. According to the Miettinen classification, eight patients (30.8%) had high risk, three (11.5%) had intermediate risk, 14 (53.8%) had low risk, and one (3.9%) had very low risk (Table 1). Based on the modified Fletcher classification, 11 patients (42.3%) had high risk, 12 (46.2%) had low risk, and three (11.5%) had very low risk. Adjuvant imatinib therapy was administered to nine patients, primarily those at high risk for disease (Figure 3).

### 3.5. Recurrence and Survival

During the follow-up period, six patients experienced recurrence; five were classified as high risk and one as low risk. In the high-risk group, several patients underwent sequential tyrosine kinase inhibitor therapy (imatinib, sunitinib, and regorafenib) and local interventions. Two patients died of disease; one at 10 years and 4 months and one at 15 years and 10 months after initial surgery. The patient with low risk was classified as low risk using both the Miettinen and modified Fletcher systems and had a tumor measuring 45 mm with a mitotic index of 3 per 50 HPFs and Ki-67 of 10%. This patient exhibited partial resistance to imatinib and required multimodal treatment, including repeat surgery.

**Figure 4:**
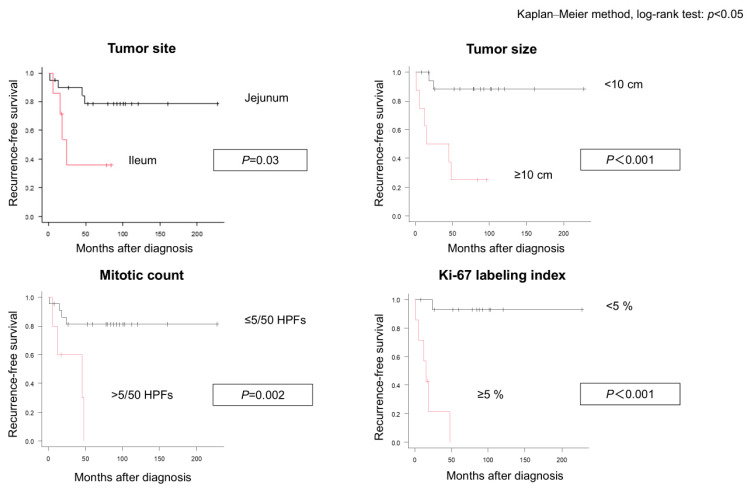
Kaplan–Meier analysis of recurrence-free survival. Kaplan–Meier curves showing recurrence-free survival stratified by tumor location (jejunum vs. ileum), tumor size (<10 cm vs. ≥10 cm), mitotic index (≤5 vs. >5/50 HPFs), and Ki-67 labeling index (<5% vs. ≥5%). HPFs, high-power fields.

The Kaplan–Meier analysis revealed a 5-year OS rate of 91.3% and RFS rate of 68.7%. OS did not differ significantly according to age, sex, or symptom status. However, RFS was significantly worse in patients with ileal tumors (35.7% vs. 78.5%, *p* = 0.03), tumor size ≥ 10 cm (25.0% vs. 88.2%, *p* < 0.001), mitotic index > 5/50 HPFs (*p* = 0.002), and Ki-67 labeling index ≥ 5% (*p* < 0.001) (Table 2 and Figure 4). Risk classification using the Miettinen and modified Fletcher classifications also correlated with RFS. The sensitivity analysis assuming missing cases as <5% or ≥5% did not alter the direction of the association. In multivariate Cox analysis restricted to complete cases for Ki-67 (*n* = 22, seven events), Ki-67 ≥ 5% remained a significant predictor of inferior RFS (HR, 21.22; 95% CI, 1.58–284.06; *p* = 0.021), whereas tumor size ≥ 10 cm did not reach statistical significance (HR, 2.01; 95% CI, 0.27–14.78; *p* = 0.494). In the sensitivity model, adding ileal location, Ki-67 ≥ 5% remained significant (HR, 19.32; 95% CI, 1.75–213.06; *p* = 0.016).

## 4. Discussion

This single-center cohort study highlights three clinically relevant points for small bowel gastrointestinal stromal tumors (GISTs): (1) the persistent limitation of preoperative histological confirmation despite contemporary enteroscopic techniques, (2) the distinctive phenotype of NF1-associated small bowel GIST, and (3) the prognostic contribution of the Ki-67 labeling index beyond conventional risk models. As the median follow-up exceeded a decade, our data also documented late recurrences beyond 10 years, which underscores the need to revisit surveillance paradigms for selected patients.

### 4.1. Diagnostic Challenges and Limited Yields of Enteroscopic Biopsy

Although capsule- and balloon-assisted enteroscopy have transformed visualization of small bowel pathology, their biopsy yields for submucosal GISTs remain modest in practice. In our case series, histological confirmation via enteroscopic biopsy was achieved in one of six biopsied cases, which is consistent with prior studies showing incomplete procedures and limited diagnostic impact in subepithelial lesions [3,4]. Given the bleeding risk and the predominantly extraluminal growth pattern, our findings reinforce the use of a pragmatic approach: prioritize cross-sectional imaging for staging and plan surgical resection for both diagnosis and treatment when radiology strongly suggests GIST [1,2,12]. This addresses the common real-world gap between technical feasibility and clinical yield.

### 4.2. NF1-Associated Small Bowel GIST: A Distinct Clinical Subset

NF1 was uniformly present in 11.1% patients in our cohort, all of whom had multiple jejunal tumors. This aligns with the recognized NF1 GIST phenotype, often the KIT/PDGFRA wild-type, multiple, small bowel-predominant, and biologically distinct from sporadic disease [13]. Notably, despite intermediate or high-risk categorization according to standard systems, the long-term outcomes for our patients with NF1 were not associated with recurrence in this small cohort. This divergence suggests that anatomical site, multiplicity, and genotype interact in ways not fully characterized using the current risk tools that include factor size, site, and mitotic index alone.

Therapeutically, the variable sensitivity of NF1-GIST to imatinib (in the absence of KIT/PDGFRA mutations) highlights the need for genotype-informed management, judicious use of TKIs, and careful surgical planning for multifocal disease [5,13].

NF1-associated GISTs showed multiple jejunal tumors in our cohort, consistent with prior reports. However, given the small number of cases (*n* = 3), these observations are preliminary and hypothesis-generating rather than definitive.

### 4.3. Ki-67 Labeling Index Refined Risk Beyond Conventional Models

Although tumor size, site, and mitotic activity form the backbone of the Miettinen and modified Fletcher classifications, proliferative indices offer complementary granularity. We found Ki-67 ≥ 5% to be associated with inferior RFS, alongside ileal location, tumor size ≥ 10 cm, and mitoses > 5/50 HPFs. Although this aligns with meta-analytic evidence that Ki-67 correlates with adverse outcomes in GIST [7,8,14], as well as with recent findings demonstrating the correlation of Ki-67 with tumor size in cases of small bowel tumors [15], our study is notable for having been small bowel-specific with long-term follow-up. Importantly, a patient categorized as having low-risk experienced recurrence with 10% Ki-67, which illustrates how proliferation metrics can unmask residual risk insufficiently obtained by mitotic counts alone.

Two key implications emerged from this study. First, Ki-67 can serve as a practical adjunct in postoperative risk communication and tailoring surveillance methods. Second, our data motivate the prospective validation of a hybrid model (site + size + mitoses + Ki-67), particularly for small bowel GIST when malignant potential exceeds that of gastric primaries. We caution that our analysis was univariate; hence, we have interpreted Ki-67 as a refining rather than an independent factor, pending research with larger, multivariable datasets. The worse prognosis observed in ileal GISTs may reflect site-specific microenvironmental factors such as differences in vascular supply, immune surveillance, and exposure to bile acids [9]. Emerging molecular data suggest that ileal tumors may harbor distinct KIT or PDGFRA mutation patterns and alternative signaling pathways, which could contribute to their aggressive phenotype [16]. A high Ki-67 labeling index indicates increased proliferative activity, which is biologically linked to genomic instability and accelerated tumor progression [8]. This proliferative marker may capture dynamic aspects of tumor biology that are not fully elucidated by static mitotic counts, supporting its role as a complementary prognostic factor. Furthermore, NF1-related GISTs involve dysregulation of the RAS/MAPK pathway, which explains their multifocal presentation and distinct clinical behavior [13]. Microenvironmental factors such as bile acid exposure, vascular supply, and immune surveillance may contribute to the aggressive phenotype of ileal tumors [9,17]. Tumor dormancy and metastatic latency, mediated by quiescent cell states under immune or metabolic stress, provide a plausible explanation for very late recurrences observed in our cohort (>10 years) [18,19]. These biological insights align with our clinical finding of Ki-67 ≥ 5% as a predictor of adverse outcomes, supporting its role as a surrogate for proliferative signaling beyond the mitotic index. This clinical observation underscores the fact that even tumors categorized as low risk may harbor residual malignant potential when Ki-67 is elevated, thus supporting the role of Ki-67 as a complementary marker for refining surveillance strategies. Taken together, recent meta-analyses and molecular studies corroborate these associations [7,8,14,18].

### 4.4. Late Recurrence and Surveillance: Are Current Schedules Sufficient?

We observed recurrences, including very late events (>10 years) and two disease-specific deaths at 10 years and 4 months and 15 years and 10 months post-resection, respectively. Although late recurrence beyond 10 years is uncommon, it has been documented in large pooled analyses [20], supporting our observation of very late events. Contemporary guidance endorses risk-adapted imaging intervals and, for low-risk disease, de-escalation over time [1,12]; a recent study with a low-risk cohort even questions the value of intensive surveillance [21]. Our small bowel-focused data suggest a nuanced message: site matters, and even nominally low-risk small bowel tumors—with adverse biology flagged by Ki-67—may warrant extended, lower-intensity surveillance beyond 5 years. This proposition remains hypothesis-generating, although it is clinically actionable and consistent with the higher baseline aggressiveness of small bowel GIST. Even patients categorized as low risk may require prolonged, low-intensity follow-up when Ki-67 ≥ 5% or ileal location is present. Furthermore, we compared our findings with a large cohort study that reported the recurrence rate decreased from 70.5% at 5 years to 62.9% at 10 years, which is a reduction of only 7.6% [20]. Although this absolute risk reduction beyond 5 years is modest, our observation of very late events (>10 years) in patients with small bowel GISTs suggests that extended, low-intensity surveillance may still be reasonable for selected patients. This recommendation should be considered hypothesis-generating rather than definitive. Our data suggest that extended, low-intensity surveillance beyond 5 years may be reasonable for selected patients, particularly those with ileal tumors or Ki-67 ≥ 5%. This recommendation is also hypothesis-generating and should be balanced against recent literature such as the study by Joensuu et al. [20], which reported only modest risk reduction beyond 5 years. Very late recurrence (>10 years) may be explained by tumor dormancy and micro-metastatic persistence [14]. Dormant GIST cells can remain in a quiescent state under immune control or nutrient limitation and reactivate later due to changes in the microenvironment or host immunity [18]. This concept aligns with the theories of metastatic latency observed in other sarcomas. Recent evidence further supports these concepts. Kirkland (2023) comprehensively reviewed the mechanisms underlying tumor dormancy and disease recurrence, highlighting how quiescent cancer cells under metabolic or immune stress may persist for years and later reactivate [19]. These insights provide a biological rationale for our observation of very late recurrences and reinforce the need for prolonged, risk-adapted surveillance in cases of small bowel GISTs.

### 4.5. Treatment Courses and Outcomes in Context

The 5-year OS rate was 91.3% and the RFS rate was 68.7%, which reflects the combined impact of complete resection and availability of sequential TKIs (imatinib, sunitinib, and regorafenib) in recurrent disease [1,2,12]. The patients with recurrence in our cohort frequently required sequential tyrosine kinase inhibitor therapy (imatinib, sunitinib, and regorafenib) and local interventions, which reflects the complexity of managing advanced disease. These observations underscore the need for risk-adapted, lifelong follow-up to enable early detection and timely treatment of recurrences, particularly in ileal tumors, large primaries, high mitotic rate, and elevated Ki-67. Notably, the adjuvant imatinib therapy administered to high-risk patients may have partially influenced recurrence-free survival and reduced the apparent “natural” recurrence risk. This potential confounding effect should be considered when interpreting our findings.

### 4.6. Comparison with a Recent Single-Institutional Study

A recent Chiba University study [22] analyzed 26 small intestinal GIST cases, including 10 duodenal tumors, and identified recurrence-associated factors such as maximum standardized uptake value ≥ 8.4, Ki-67 ≥ 10%, and inflammatory markers [17]. In contrast, our study excluded duodenal tumors, focused on jejunal/ileal GISTs, and highlighted NF1-associated multiplicity, late recurrence beyond 10 years, and a prognostic value of Ki-67 ≥ 5%. Both studies support incorporating Ki-67 into risk stratification, although the cutoff values differ. Furthermore, previous large-scale population-based analyses have reported minimal differences in cause-specific and overall survival between duodenal and jejuno-ileal GISTs [23,24]. These findings contrast with our rationale for excluding duodenal tumors, which was based on their anatomical proximity to the stomach and distinct diagnostic pathways. We acknowledge this controversy and emphasize that our results should be interpreted cautiously within this broader context. This approach does not contradict the findings of large-scale studies. Duodenal GISTs differ substantially in terms of diagnostic pathways (often detected via upper endoscopy) and surgical management (e.g., pancreaticoduodenectomy), whereas jejuno-ileal GISTs typically present with delayed diagnosis, obstruction, or bleeding and follow different management strategies. These anatomical and therapeutic distinctions could confound site-specific prognostic analyses; therefore, focusing on jejuno-ileal tumors ensured a more homogeneous cohort for evaluation of biological factors such as Ki-67 and NF1-related multiplicity.

### 4.7. Strengths and Limitations

The strengths of this study include (1) a 15-year single-center follow-up focused on jejunal/ileal GIST, (2) systematic collection of enteroscopic diagnostic yield, (3) incorporation of NF1-associated cases, and (4) long follow-up enabling detection of late events.

This study has several limitations. First, the retrospective design and modest sample size limited the statistical power and generalizability. Second, the single-center setting may have introduced selection bias. Third, the NF1 diagnosis was based on clinical criteria without genetic confirmation. Although molecular confirmation is ideal, the NIH consensus clinical criteria remain widely accepted for a definite diagnosis in clinical practice [25]. Fourth, molecular profiling was incomplete; thus, the genotype–phenotype associations should be interpreted cautiously. Fifth, the Ki-67 cut-off (5%) was chosen a priori and alternative thresholds require validation in larger cohorts. In particular, the recurrence observed in a patient with a Ki-67 index of 10% despite low-risk classification highlights the need for validation of optimal cut-off values and assessment of the prognostic accuracy with higher thresholds. These limitations should be considered when interpreting the prognostic implications and surveillance recommendations proposed in this study. Finally, the small number of recurrence events may have resulted in unstable estimates in multivariable Cox regression (low events-per-variable ratio). Therefore, the results should be interpreted cautiously and validated in larger cohorts.

### 4.8. Clinical Implications

Our findings may have potential implications for surveillance strategies. While current guidelines recommend risk-adapted imaging intervals based on size, site, and mitotic index, our data suggest that Ki-67 ≥ 5% could be explored as an additional factor in future studies. For example, patients with ileal tumors or Ki-67 ≥ 5%—even if classified as low risk by conventional models—may benefit from extended follow-up beyond 5 years in which annual or biennial imaging is obtained after the initial intensive phase. Conversely, patients with jejunal tumors and Ki-67 < 5% could follow standard de-escalation schedules.

We propose that future protocols consider a hybrid risk model (site + size + mitotic index + Ki-67) to refine surveillance intensity. For NF1-associated GIST, individualized strategies are warranted, including closer monitoring for multifocal disease and genotype-informed therapeutic planning. Although these recommendations remain hypothesis-generating, they provide a framework for prospective validation and guideline development.

## 5. Conclusions

The findings of this study indicate that the Ki-67 labeling index, tumor location, and tumor size may be associated with the prognosis of small bowel GISTs. Given the small sample size and retrospective study design, these findings should be interpreted with caution. NF1-associated cases had unique clinical features, although these observations remain preliminary. Late recurrences beyond 10 years were documented, which highlights the need for individualized, risk-adapted follow-up strategies.

## Figures and Tables

**Table 1:** Baseline characteristics, diagnostic modalities, treatment, and risk classifications for 27 patients with small bowel gastrointestinal stromal tumors.

Variable	*n* (%) or Mean (Range)
Age (years)	62.2 (25–86)
Sex	Men: 11 (40.7), Women: 16 (59.3)
Tumor location	Jejunum: 20 (74.1), Ileum: 7 (25.9)
Symptoms at diagnosis	Present: 20 (74.1), Absent: 7 (25.9)
Gastrointestinal bleeding	12
Abdominal pain (including ileus)	4
Abdominal distension	3
Palpable mass	1
History of other malignancy	7 (25.9)
Neurofibromatosis type 1	3 (11.1) *
Imaging modalities	CT: 24 (88.8), MRI: 2 (7.4), Capsule endoscopy: 1 (3.7)
Balloon-assisted enteroscopy	Performed: 14 (51.9)
Biopsy attempted	6
Histological diagnosis achieved	1
Mean tumor size (mm)	62.4 (13–145)
Distant metastasis at diagnosis	2 (7.4) (Liver: 1, Peritoneum: 1)
Risk classification	
Miettinen	High: 8 (30.8), Intermediate: 3 (11.5), Low: 14 (53.8), Very low: 1 (3.9)
Modified Fletcher	High: 11 (42.3), Low: 12 (46.2), Very low: 3 (11.5)

Values are presented as mean (range) or *n* (%). CT, computed tomography; MRI, magnetic resonance imaging. * All NF1 cases had multiple jejunal tumors. Age, sex, tumor location, presenting symptoms, history of other malignancies, presence of neurofibromatosis type 1 (NF1), imaging and endoscopic procedures, tumor size, presence of metastases, surgical resection, and risk stratification by Miettinen and modified Fletcher classifications were included.

**Table 2:** Results of univariate analysis of prognostic factors for recurrence-free survival (RFS), including tumor location, size, mitotic index, Ki-67 labeling index, and risk classification.

Factor	Cases (*n*)	Recurrence Events (*n*)	5-Year OS (%)	*p*-Value	5-Year RFS (%)	*p*-Value
Age ≥ 65 years	13	1	83.9%	0.13	74.6%	0.45
Age < 65 years	14	5	100%	—	61.9%	—
Sex: Male	11	2	79.5%	0.23	57.7%	0.31
Sex: Female	16	4	100%	—	75.0%	—
Symptoms present	20	4	89.7%	0.94	68.7%	0.97
Symptoms absent	7	2	100%	—	68.6%	—
History of malignancy: Yes	7	0	83.3%	0.62	83.3%	0.34
History of malignancy: No	20	6	95.0%	—	63.2%	—
Tumor location: Jejunum	20	3	95.0%	0.94	78.5%	0.03
Tumor location: Ileum	7	3	83.3%	—	35.7%	—
Tumor size ≥ 10 cm	8	5	87.5%	0.16	25.0%	<0.001
Tumor size < 10 cm	19	1	94.1%	—	88.2%	—
Mitotic count > 5/50 HPFs	5	4	100%	0.42	NA	0.002
Mitotic count ≤ 5/50 HPFs	22	2	90.7%	—	81.1%	—
Ki-67 ≥ 5%	7	5	85.7%	0.26	NA	<0.001
Ki-67 < 5%	15	0	92.9%	—	92.9%	—
Risk classification (Miettinen): High	9	5	88.9%	0.18	27.8%	<0.001
Risk classification (Miettinen): Intermediate/Low/Very low	18	1	94.1%	—	88.2%	—
Risk classification (Modified Fletcher): High	12	5	91.7%	0.61	47.6%	0.03
Risk classification (Modified Fletcher): Intermediate/Low/Very low	15	1	92.9%	—	85.7%	—

The number of recurrence events for each subgroup is shown to enhance interpretability. OS, overall survival; RFS, recurrence-free survival; HPF, high-power field; NA, not applicable. — indicates not applicable or not calculated. *p*-values were calculated using the log-rank test.

## Data Availability

The datasets generated and/or analyzed during the current study are available from the corresponding author upon reasonable request.

## Abbreviations

The following abbreviations are used in this manuscript:

BAE	balloon-assisted enteroscopy
CT	computed tomography
GIST	gastrointestinal stromal tumor
HPFs	high-power fields
MRI	magnetic resonance imaging
NF1	neurofibromatosis type 1
OS	overall survival
RFS	recurrence-free survival
TKI	tyrosine kinase inhibitors
